# Supplementary material for: Reservoir-Style Polymeric Drug Delivery Systems: Empirical and Predictive Models for Implant Design
Source: Pharmaceuticals (Basel). 2022 Oct 3;15(10):1226. doi: 10.3390/ph15101226 (PMC9610229; doi:10.3390/ph15101226)
Supplement: Supplementary file 1 [file pharmaceuticals-15-01226-s001.zip › pharmaceuticals-1896337-supplementary.pdf]

Supplementary Materials: Reservoir-Style Polymeric Drug Delivery Systems: Empirical and Predictive Models for Implant Design

Linying Li[1], Chanhwa Lee[2], Daniela F. Cruz[1], Sai Archana Krovi[1], Michael G. Hudgens[2], Mackenzie L. Cottrell[3], Leah M. Johnson[1 *]

[1] RTI International, 3040 E. Cornwallis Road, Research Triangle Park, NC 27709

[2] Dept of Biostatistics, Gillings School of Global Public Health, University of North Carolina, Chapel Hill, NC 27599

[3] Division of Pharmacotherapy and Experimental Therapeutics, Eshelman School of Pharmacy, University of North Carolina, Chapel Hill, NC 27599

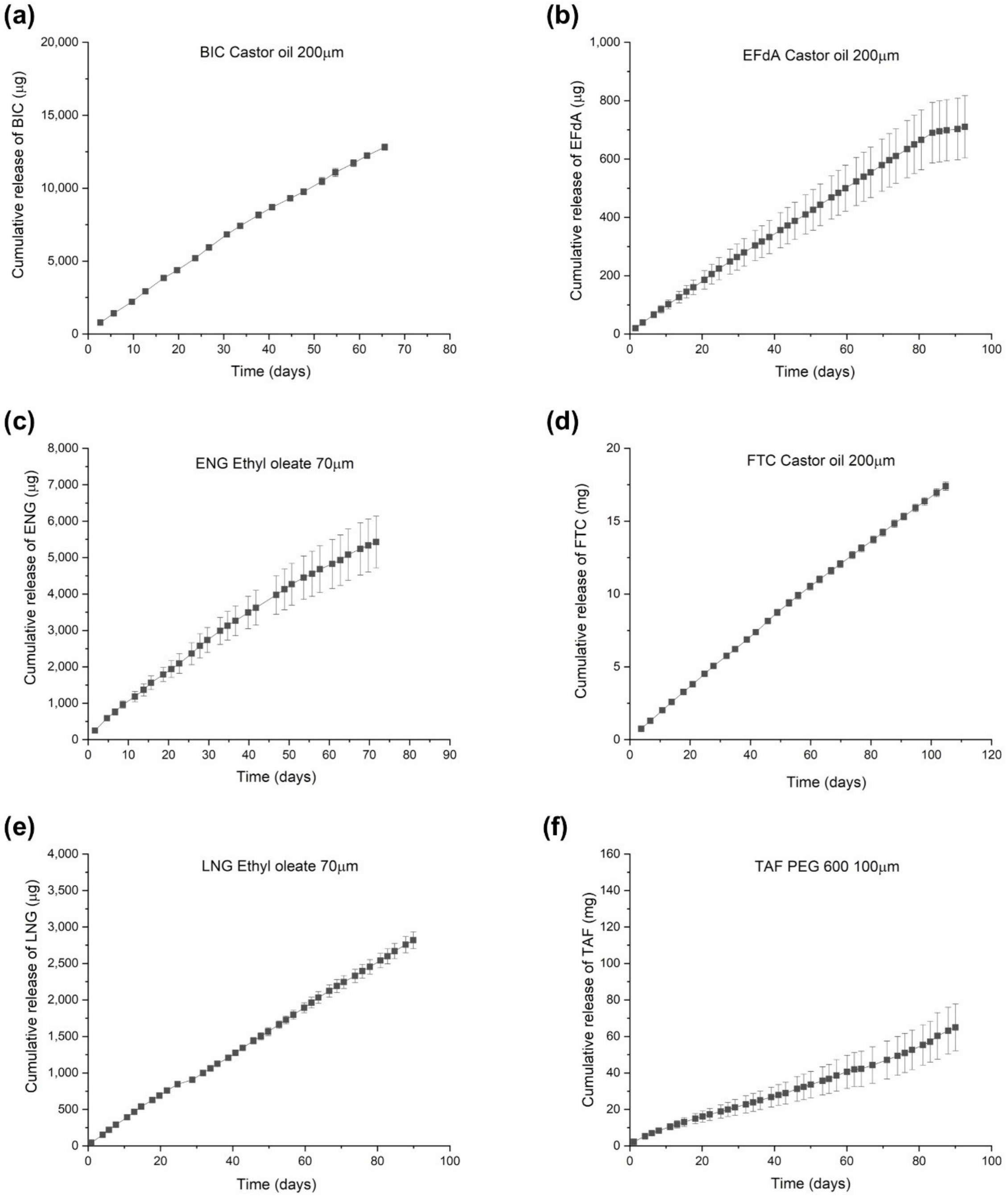

Figure S1. Cumulative release profiles of active pharmaceutical ingredients (APIs) from PCL implants with different configurations: (a) BIC (castor oil, PC-17, 40 mm length, 200 μm wall thickness, drug to excipient ratio: 2:1), (b) EFdA (castor oil, PC-17, 10 mm length, 200 μm wall thickness, drug to excipient ratio: 1:1), (c) ENG (ethyl oleate, Sigma, 10 mm length, 70 μm wall

thickness, drug to excipient ratio: 2:1), (d) FTC (castor oil, PC-17, 40 mm length, 200 μm wall thickness, drug to excipient ratio: 1:1), (e) LNG (ethyl oleate, Sigma, 10 mm length, 70 μm wall thickness, drug to excipient ratio: 2:1), and (f) TAF_{salt} (PEG 600, Sigma, 40 mm length, 100 μm wall thickness, drug to excipient ratio: 2:1).

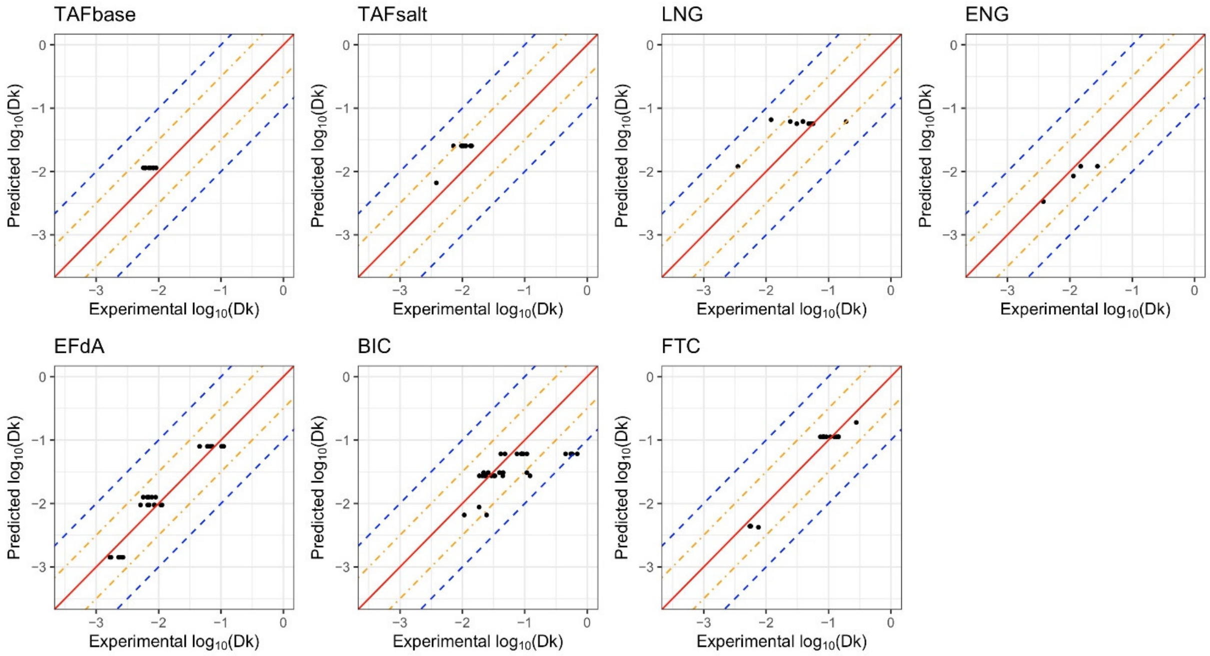

Figure S2. Predicted and experimental values of $log_{10}(\boldsymbol{Dk})$ by API. The solid red diagonal line indicates when the prediction and observation are the same, the orange and blue dashed lines indicate predictive values within 0.5 log and 1 log of the observed values, respectively.

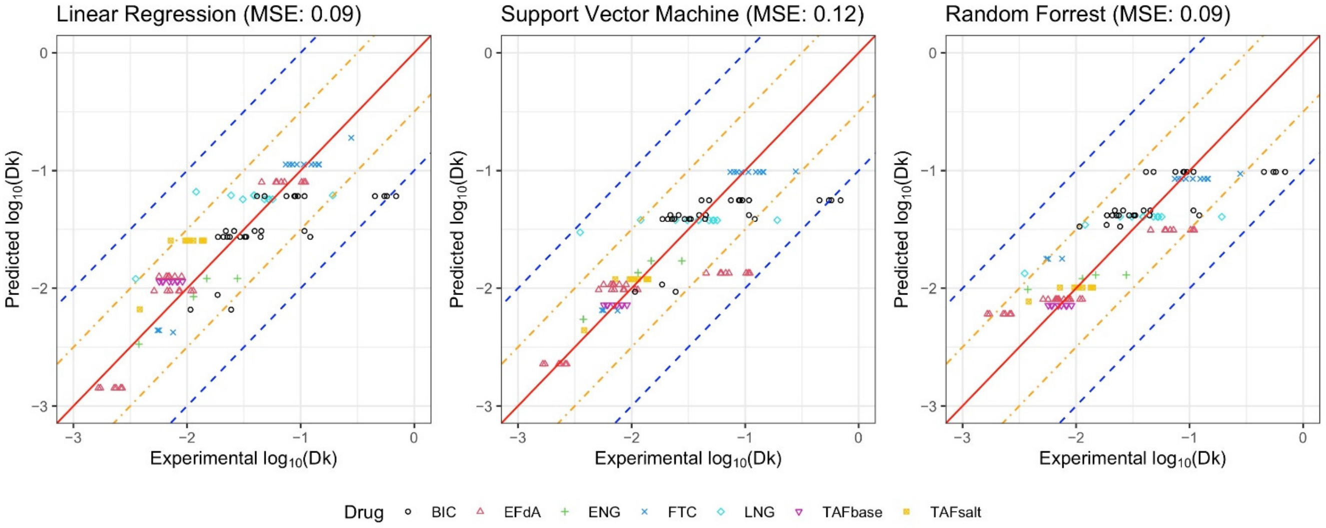

Figure S3. Comparison of the linear prediction model and machine learning models. The solid red diagonal line indicates when the prediction and observation are the same, the orange and blue dashed lines indicate predictive values within 0.5 log and 1 log of the observed values, respectively.

Table S1. The predicted and experimental values for Dk and daily release rates for the implants containing different drug formulations.

API	Excipients	PCL type	PCL wall thickness (μm)	Length of the implant (mm)	Experimental Dk (mm²/day)	Predicted Dk (mm²/day)	Experimental release rate (mg/day)	Predicted release rate (mg/day)
TAF_{salt}	Castor oil	Sigma	70	40	1.41E-02	1.83E-02	0.75	1.016
		Sigma	100	40	1.14E-02	1.83E-02	0.43	0.711
		Sigma	200	40	1.05E-02	1.83E-02	0.19	0.355
		Sigma	70	40	1.37E-02	1.83E-02	0.74	1.016
		Sigma	70	40	1.35E-02	1.83E-02	0.72	1.016
		Sigma	100	40	9.65E-03	1.83E-02	0.36	0.711
		Sigma	100	40	7.19E-03	1.83E-02	0.26	0.711
	PEG 600	Sigma	100	40	3.83E-03	4.54E-03	0.64	0.821
	Castor oil	Sigma	100	40	9.98E-03	1.83E-02	0.16	0.711
TAF_{base}	Castor oil	Sigma	100	40	6.15E-03	1.63E-02	0.24	0.845
		Sigma	150	40	6.97E-03	1.63E-02	0.17	0.564
		Sigma	200	40	7.42E-03	1.63E-02	0.14	0.423
		Sigma	45	40	9.11E-03	1.63E-02	0.78	1.879
		Sigma	70	40	8.28E-03	1.63E-02	0.44	1.208
		PC-17	150	40	8.15E-03	1.63E-02	0.27	1.208
		PC-17	300	40	5.70E-03	1.63E-02	0.09	0.564
LNG	Ethyl Oleate	Sigma	70	10	4.81E-02	4.81E-02	0.031	0.282
	Sesame Oil	Sigma	70	10	3.91E-02	5.22E-02	0.023	0.032
	Oleic Acid	Sigma	70	10	1.20E-02	5.59E-02	0.007	0.032
	Propylene glycol	Sigma	70	10	3.53E-03	9.61E-03	0.013	0.031
	Ethyl Oleate	PC-12	70	10	3.10E-02	4.81E-02	0.021	0.038
	Sesame oil	PC-12	70	10	2.44E-02	5.22E-02	0.015	0.032
	Sesame oil	PC-17	150	25	3.87E-02	5.22E-02	0.027	0.032
	Ethyl oleate	PC-17	100	13	5.29E-02	4.81E-02	0.032	0.037
	Ethyl oleate	PC-17	150	17	5.67E-02	4.81E-02	0.029	0.029
	Ethyl oleate	PC-17	200	20	5.21E-02	4.81E-02	0.024	0.025
	Sesame oil	PC-17	300	12	1.92E-01	5.22E-02	0.030	0.022
ENG	Ethyl oleate	Sigma	70	10	1.14E-02	1.12E-02	0.071	0.009
	Sesame oil	Sigma	70	10	1.49E-02	1.61E-02	0.061	0.070
	Castor oil	Sigma	70	10	3.76E-03	4.27E-03	0.067	0.068
	Sesame oil	PC-17	300	12	2.77E-02	1.61E-02	0.030	0.078
EFdA	Sesame oil	PC-17	100	10	1.10E-01	8.34E-02	0.0191	0.019
	Sesame oil	PC-17	150	10	6.18E-02	8.34E-02	0.0068	0.014
	Sesame oil	PC-17	200	10	7.20E-02	8.34E-02	0.0059	0.010
	Castor oil	PC-17	100	10	1.08E-02	9.21E-03	0.0212	0.007

	Castor oil	PC-17	150	10	7.05E-03	9.21E-03	0.0089	0.018
	Castor oil	PC-17	200	10	8.46E-03	9.21E-03	0.0078	0.012
	Sesame oil	PC-17	100	10	1.02E-01	8.34E-02	0.0168	0.009
	Castor oil	PC-17	100	10	1.13E-02	9.21E-03	0.0144	0.014
	Castor oil	PC-17	150	10	6.68E-03	9.21E-03	0.0062	0.018
	Castor oil	PC-17	200	10	8.64E-03	9.21E-03	0.0058	0.012
	Castor oil	PC-17	300	10	5.13E-03	9.21E-03	0.0023	0.009
	Sesame oil	PC-17	100	10	1.07E-01	8.34E-02	0.0173	0.006
	Sesame oil	PC-17	150	10	6.05E-02	8.34E-02	0.0064	0.014
	Sesame oil	PC-17	200	10	6.74E-02	8.34E-02	0.0052	0.010
	Sesame oil	PC-17	300	10	4.54E-02	8.34E-02	0.0022	0.007
	Castor oil	PC-31	150	10	6.83E-03	1.23E-02	0.0062	0.005
	Castor oil	PC-31	200	10	6.57E-03	1.23E-02	0.0043	0.012
	Castor oil	PC-41	150	10	7.80E-03	1.23E-02	0.0070	0.009
	Castor oil	PC-41	200	10	5.67E-03	1.23E-02	0.0037	0.012
	Glycerol	PC-31	150	10	2.29E-03	1.29E-03	0.0130	0.009
	Glycerol	PC-31	200	10	2.61E-03	1.29E-03	0.0108	0.015
	Glycerol	PC-41	150	10	1.66E-03	1.29E-03	0.0092	0.011
	Glycerol	PC-41	200	10	1.73E-03	1.29E-03	0.0071	0.015
	Castor oil	PC-31	300	10	8.95E-03	1.23E-02	0.0037	0.011
	Castor oil	PC-41	300	10	6.97E-03	1.23E-02	0.0029	0.006
	Glycerol	PC-31	300	10	2.68E-03	1.29E-03	0.0072	0.006
	Glycerol	PC-41	300	10	2.37E-03	1.29E-03	0.0062	0.007
BIC	Sesame oil	PC-17	100	40	5.75E-01	6.49E-02	0.725	0.007
	Sesame oil	PC-17	150	40	5.46E-01	6.49E-02	0.449	0.356
	Sesame oil	PC-17	200	40	6.93E-01	6.49E-02	0.418	0.238
	Sesame oil	PC-17	300	40	4.51E-01	6.49E-02	0.173	0.178
	Glycerol	PC-17	100	40	1.08E-01	3.20E-02	0.629	0.119
	Castor oil	PC-17	100	40	1.22E-01	2.84E-02	0.956	0.383
	PEG 40 Castor oil	PC-17	100	40	2.44E-02	6.51E-03	0.939	0.388
	Oleic acid	PC-17	100	40	1.86E-02	8.77E-03	0.521	0.452
	Sesame oil	PC-41	150	40	8.80E-02	6.49E-02	0.298	0.438
	Castor oil	PC-41	150	40	3.32E-02	2.84E-02	0.287	0.238
	Sesame oil	PC-41	200	40	8.93E-02	6.49E-02	0.224	0.259
	Castor oil	PC-41	200	40	3.22E-02	2.84E-02	0.201	0.178
	Sesame oil	PC-17	200	40	4.13E-02	6.49E-02	0.301	0.194
	Glycerol	PC-31	150	40	3.94E-02	3.20E-02	0.296	0.178
	Castor oil	PC-31	150	40	4.44E-02	2.84E-02	0.378	0.255
	PEG 40 Castor oil	PC-31	150	40	1.07E-02	6.51E-03	0.476	0.259
	Sesame oil	PC-31	150	40	9.45E-02	6.49E-02	0.321	0.301
	Sesame oil	PC-17	200	10	1.08E-01	6.49E-02	0.073	0.238
	Sesame oil	PC-31	200	10	8.96E-02	6.49E-02	0.062	0.045
	Sesame oil	PC-41	200	10	7.49E-02	6.49E-02	0.050	0.045
	Sesame oil	PC-17	300	40	4.81E-02	6.49E-02	0.074	0.045

	Castor oil	PC-17	300	40	1.87E-02	2.84E-02	0.070	0.119
	Castor oil	PC-17	150	40	2.38E-02	2.84E-02	0.218	0.129
	Castor oil	PC-17	200	40	2.93E-02	2.84E-02	0.191	0.259
	Castor oil	PC-17	250	40	2.27E-02	2.84E-02	0.113	0.194
	Castor oil	PC-17	300	40	2.09E-02	2.84E-02	0.084	0.155
	Glycerol	PC-17	100	40	4.50E-02	3.20E-02	0.487	0.129
	Glycerol	PC-17	200	40	2.60E-02	3.20E-02	0.136	0.383
	Glycerol	PC-17	300	40	2.20E-02	3.20E-02	0.074	0.192
FTC	Castor oil	PC-17	100	40	1.45E-01	1.16E-01	0.40	0.128
	Castor oil	PC-17	200	40	1.07E-01	1.16E-01	0.14	0.331
	Castor oil	PC-17	300	40	7.40E-02	1.16E-01	0.06	0.166
	Glycerol	PC-17	300	40	5.49E-03	4.04E-03	0.19	0.110
	Castor oil	PC-31	150	40	8.45E-02	1.16E-01	0.19	0.156
	Oleic acid	PC-31	150	40	2.79E-01	2.00E-01	0.36	0.221
	Propylene glycol	PC-31	300	40	7.52E-03	3.88E-03	0.28	0.209
	Castor oil	PC-17	150	40	8.47E-02	1.16E-01	0.19	0.157
	Glycerol	PC-17	300	40	5.67E-03	4.04E-03	0.21	0.221
	Castor oil	PC-41	150	40	9.25E-02	1.16E-01	0.17	0.156
	Castor oil	PC-41	200	40	7.98E-02	1.16E-01	0.11	0.221
	Castor oil	PC-17	100	40	1.43E-01	1.16E-01	0.39	0.166
	Castor oil	PC-17	200	40	1.24E-01	1.16E-01	0.17	0.331
	Castor oil	PC-17	200	40	1.34E-01	1.16E-01	0.18	0.166

Table S2. The solubility of the API within various pharmaceutical grade excipients.

Excipient	3TC solubility (mg/mL)	ABC Solubility (mg/mL)	RAL potassium Solubility (mg/mL)	DTG sodium solubility (mg/mL)
Castor Oil	0.37 ± 0.001	9.44 ± 1.46	0.061 ± 0.008	0.08 ± 0.01
Cottonseed Oil	0.004 ± 0.003	0.837 ± 0.62	0.006 ± 0.001	0.06 ± 0.02
Ethyl Oleate	0.004 ± 0.001	0.066 ± 0.003	0.004 ± 0.001	0.04 ± 0.03
Glycerol	121.08 ± 19.0	27.50 ± 13.87	30.61 ± 2.48	3.57 ± 0.002
Oleic Acid	1.13 ± 0.001	52.68 ± 1.35	0.20 ± 0.009	2.48 ± 0.22
PEG$_{300}$	72.98 ± 0.31	72.46 ± 4.31	56.9 ± 3.00	1.75 ± 0.11
PEG$_{400}$	55.16 ± 0.19	75.84 ± 0.72	33.6 ± 1.96	0.95 ± 0.05
PEG$_{600}$	41.14 ± 0.94	73.00 ± 4.50	18.6 ± 1.37	0.61 ± 0.03
PEG$_{40}$ Castor Oil	8.13 ± 0.004	21.85 ± 0.70	6.16 ± 0.012	1.91 ± 0.15
Polysorbate 80	6.83 ± 0.77	22.65 ± 0.87	2.10 ± 0.26	1.34 ± 0.63
Propylene Glycol	0.02 ± 0.008	71.56 ± 0.16	0.004 ± 0.001	0.02 ± 0.002
Sesame Oil	0.004 ± 0.003	0.149 ± 0.01	0.008 ± 0.003	0.03 ± 0.01
